# The Perception of Medical Students at King Abdulaziz University Hospital Regarding the Liver Transplant Allocation System

**DOI:** 10.7759/cureus.6187

**Published:** 2019-11-18

**Authors:** Mohammed A Safhi, Mohammed Alzahrani, Khaled W Altahini, Abdulaziz Kilfaden, Abdulrahman A Bagber, Mohammed R Algethami, Wisam Jamal, Hisham Rizk

**Affiliations:** 1 Surgery, Faculty of Medicine, King Abdulaziz University, Jeddah, SAU; 2 Internal Medicine, Faculty of Medicine, King Abdulaziz University, Jeddah, SAU; 3 Preventive Medicine, Joint Program, Aseer Region, Jeddah, SAU; 4 General Surgery, Faculty of Medicine, University of Jeddah, Jeddah, SAU

**Keywords:** quality of life, prospect of success, liver transplantation, allocation, benefit, urgency, willingness to donate

## Abstract

Background: The benefit of liver transplantation is not only to increase the patient’s lifetime but also for persistent relief of pain and anxiety. Shortage of the organ is the main hindrance of transplantation around the world, leading authorities to pass a general law for the reasonable distribution of organs and come up with the Model for End-Stage Liver Disease (MELD) system which scores the severity of liver disease and risk of mortality in order to detect the mechanism of allocation.

Objective: This study aims to assess medical students’ perception of the liver transplant and allocation system.

Methods: A cross-sectional survey was carried out among 402 medical students at King Abdulaziz University in Jeddah, Saudi Arabia.

Results: The majority of the medical students (84.4%) believed that a successful liver transplant improves both lifetime and quality of life. Most of the students also saw that the minimum survival rate should be five years after transplantation and that the patient should recover to be at least ambulatory, even if restricted by strenuous physical activity. When asked whether urgency or prospect of success defined a successful transplant, most of the students who chose urgency were preclinical (50.7%), while the prospect of success was the dominant answer chosen by students in their clinical years of study (66.1%).

Conclusion: The criteria determining the success of a liver transplant include a gain in both lifetime and quality of life. The majority of respondents wanted the capacity to benefit to be considered in the liver allocation system.

## Introduction

Liver transplantation is a surgical procedure accomplished to help replace a diseased part of a liver with a healthy one from a different individual. The donated part can be taken from a deceased or a living person [1]. Orthotopic liver transplantation (OLT) is the most common type of liver transplant used worldwide [2]. It is one of the most successful treatments of end-stage liver failure [3]. In 2018, the liver was ranked the second most common donated organ after the kidney, with 8,250 transplants according to the United Network for Organ Sharing (UNOS) [4]. In Saudi Arabia, more than 2000 liver transplants have been performed since 1990 [5].

In the United States, infection with hepatitis C virus (HCV) and hepatocellular carcinoma account for the most common indications for liver transplant [6]. On the other hand, nonalcoholic fatty liver disease is the most common indication in developed countries [7-8]. And in Saudi Arabia, a local study found that infections with HCV and hepatitis B virus are the most frequent indications for liver transplantation [5].

Because decisions involving life and death often arise with regard to organ allocation, the latter must be based on reasonable medical and ethical grounds. Liver donor allocation nowadays follows the Model for End-Stage Liver Disease (MELD), which relies on an algorithm targeting urgency, or a high-risk patient approach [9]. This system has a score ranging from 6 to 40 based on laboratory tests such as total bilirubin, serum creatinine, and the INR and it reduces mortality for patients on the transplantation waiting list [10].

A recent study conducted in Germany among medical students and other personnel, through a questionnaire about the allocation protocol, found that the benefit from organ donation outweighs the urgency and sickest-first approach [9].

Despite many studies being published on the subject, none were conducted on undergraduate medical students or in Saudi Arabia, which encouraged us to take on this research. This study aims to assess medical students’ perception of the liver transplant allocation system.

## Materials and methods

 Research design and setting

This is an observational cross-sectional study that was conducted among 402 medical students at the Faculty of Medicine of King Abdulaziz University Hospital (KAUH), Jeddah, Saudi Arabia, during the academic year 2017-2018.

 Sampling procedure

The medical bachelor program at King Abdulaziz University Faculty of Medicine is composed of two years of basic medical sciences and three years of clinical sciences. According to this, we divided the participants into two groups: preclinical and clinical.

 Measurement tools and data collection

A standardized, confidential, self-administered questionnaire was used. The questionnaire was designed using Google Forms (Mountain View, CA, USA). Questionnaire distribution and collection of response data were performed using Google spreadsheets. The survey was sent by Telegram and WhatsApp messages on the 26th of June, 2018, and responses were collected on the 26th of July, 2018. The anonymity of the respondents was preserved.

The questionnaire consisted of seven parts. The first part inquired about demographic information and academic year; the second assessed the students’ perception of the criteria for a successful liver transplant. The third part inquired about the current liver allocation system, the fourth about the implemented waiting list. The fifth part evaluated their perception of the prospect of success vs. urgency. The sixth part asked about their willingness to donate, and the last part was about alcohol-related liver cirrhosis.

 Analysis

Data were entered into Microsoft Excel 2016, and statistical analysis was performed with IBM SPSS version 21 (IBM, Armonk, NY, USA). Statistical difference between the qualitative variables was found using the chi-square test, any p-value below (0.05), CI (95%) was considered significant.

## Results

 Demographics

A total of 402 students were included (54.5% men and 49.4% women). The mean (SD) age of the participants was 21.81 (1.5) years. The students’ characteristics are shown in Table 1. 

**Table 1:** Demographic data.

		Preclinical no.	Clinical no.
Mean age (years)		20.5	22.8
Sex	Male	82	137
	Female	89	94
	Total	171	231
Smoking	Yes	25	41
	No	146	190
Body mass index	< 18.5	17	17
	18.5–24.9	94	116
	25–29.9	40	51
	≥ 30	20	45
Willingness to donate organs	Yes	65	100
	No	29	46
	Do not know	77	85

 Definition of successful liver transplantation

When asked about the criteria defining a successful liver transplantation, most of the respondents believed that success is defined as a gain in both lifetime and quality of life (84.8%). However, 8% chose “gain in quality of life” alone, and 7% “gain in a lifetime” alone. We asked students how long a patient should survive, at a minimum, after a liver transplant in order to consider it successful. Responses included “at least 5 years or longer”, chosen by 34.32% of respondents; “at least 10 years or longer” (24.62%); “at least one year or longer” (16.7%); “at least 3 months or longer” (11.9%); “at least several days or longer” (7.2%), and “at least several hours or longer” which was chosen by 5.2% of the students. Concerning improved quality of life as a criterion of successful liver transplantation, the students were asked “Which quality of life regarding independence and mobility would you expect at a minimum after transplantation in order to call it successful?” In response, 40.1% accepted that the patient be “restricted in physically strenuous activity but ambulatory and able to carry out work of a light or sedentary nature, e.g., light housework, office work,” 34.7% thought the patient should be “fully active, able to carry on all performance without restriction,” 18.8% thought they should be “ambulatory and capable of all self-care but unable to carry out any work activities, up and about more than 50% of waking hours.” Also, 5.4% chose “capable of only limited self-care, confined to bed or chair more than 50% of waking hours,” and 1% chose “completely disabled, cannot carry on any self-care, confined to bed or chair.”

 The current liver allocation system

Study participants who thought that the current allocation system for liver transplantation is fair represented 65.9%, while 34.1% considered it to be unfair. Also, we asked participants who should have a say in determining the rules of organ allocation among three groups “physicians and/or patients,” “physicians and/or society,” and “patients and/or society.” In each group, physicians represented the transplant physicians or experts; patients were defined as the affected patients via patient’s representatives; and the society represented the politics, ethics committees, faith communities, and legislature. The results gave rise to five categories; the student was either neutral between the two members, or either showed a tendency towards or chose one (Table 2).

**Table 2:** Participants’ perception on who should determine the rules of organ allocation. Physicians represents the transplant physicians or experts; patients, is defined as the affected patients via patient’s representatives; and the society represents politics, ethics commissions, faith communities, and legislature.

	Frequency	Percent
Physicians or patients		
Patients	17	4.20%
Tendency to patients	37	9.20%
Neutral	71	17.70%
Tendency to physicians	138	34.30%
Physicians	139	34.30%
Physicians or society		
Society	9	2.20%
Tendency to society	35	8.70%
Neutral	68	16.90%
Tendency to physicians	144	35.80%
Physicians	146	36.30%
Patients or society		
Society	25	6.20%
Tendency to society	62	15.40%
Neutral	100	24.90%
Tendency to patients	110	27.40%
Patients	105	26.10%
Total	402	100.00%

 The implemented “Waiting List”

As organs for donation are limited, not all patients who need a liver transplant can receive one. We asked the participants what they thought the chances were of a patient on the waiting list to receive a donor organ on time, 34.6% felt it was 40%-60%; 25.9% estimated it to be 20%-40%; 22.1% chose 60%-80%, 8.7% thought it was 80%-100%, and 8.7% estimated that there is a 0%-20% chance the patient would receive a transplantation on time. Regarding the shortage of organs, we asked the participants “As a patient on the waiting list, at what probability of dying within one year after transplantation would you accept a rejection?”; 30.1% agreed to 60%-80%; 28.4% chose 40%-60%; 16.4% chose 80%-100%; 13.2 chose 20%-40%, and 11.9% said they would accept the rejection if the probability of death was 0%-20%. 

 The prospect of success vs. urgency

We asked the participants whether the urgency of the case or the prospect of success of the transplantation should determine who receives the organ. Most of the students who chose urgency were in their preclinical years of medical school (50.7%), while the prospect of success was the dominant choice in the clinical years' students (66.1%), see Table 3 for all results. We found a statistically significant difference between the two criteria and the academic year (chi-square test value = 7.701, p = 0.021).

**Table 3:** Who should receive the organs; urgency vs. prospect of success. Participants' thoughts on determining who receives the organ, urgency of the case, or the prospect of success of the transplantation.

	Prospect of success		Neutral		Urgency		Total
	Frequency	Percent	Frequency	Percent	Frequency	Percent	
Preclinical	43	33.9%	58	42.3%	70	50.7%	171
Clinical	84	66.1%	79	57.7%	68	49.3%	231
Total	127	100%	137	100%	138	100%	402

 Willingness to donate

Participants were asked how much they were willing to help patients by donating their organs after death. The results showed that 165 (41%) of the students were willing to donate, 162 (40.3%) did not know, and 75 (18.7%) said they would not donate. Moreover, they were asked if their decision to donate was affected by the prospect of success of the transplantation or not. Most of them denied the effect (63.1%), and 36.1% agreed that the prospect of success of transplantation affected their decision to donate. The respondents were asked how the prospect of success of the transplantation affected their decision. Most of them answered that they would like to donate their organs only if given to patients with a high prospect of success (47.4%); 30.7% said they would rather donate if their organ would be given to a patient with a high prospect of success; 11.7% said they would rather donate their organs if they would be given to patients with high urgency; and 10.2% would like to donate only if their organs would be given to patients with high urgency.

 Alcohol-related liver cirrhosis

Regarding the students’ opinions on how to handle patients with alcohol-related cirrhosis, 57,7% agreed that they would accept them on the waiting list after having been abstinent for six months; 19,4% said they would never accept these patients, and 22,9% said they would always accept a patient on the waiting list.

## Discussion

In this study, most respondents agreed that the success of a liver transplant is defined as gaining in both lifetime and quality of life (84.8%). This result coincides with a recent study done in Germany [9]. The resemblance in results from two different populations leads us to perceive that the two criteria are almost equally important. Studies have also proven that liver transplantation is beneficial in improving the functioning of patients in different areas [11-13].

After asking the participants about survival as a separate criterion of liver transplant success, most respondents chose a “gain in lifetime at least five years or longer” as the determinant of liver transplant success (34.32%). “Gain in lifetime at least ten years or longer” was chosen by 24.62%. A study revealed that 43.1% of respondents from medical staff chose “gain in lifetime at least five years or longer” as the determinant of a successful transplant. In contrast, the most common interval chosen by nonmedical persons was “survival of 1 year after a liver transplant,” (41.7%). This variation could be due to the medical students’ knowledge regarding the beneficence of liver transplantation. Moreover, a prospective cohort study showed that in the last 10 years the survival rate of liver transplantation has improved: the one-year survival reached 92%, and the five-year survival reached 72%. Thus, over time, the mortality has significantly decreased [14].

On the other hand, if we considered improvement of quality of life as a criterion determining a successful liver transplant, with regard to independence and mobility, 40.1% of the respondents chose “physically restricted,” and 34.7% chose being “fully active.” In another study, most respondents found that being ambulatory and capable of all self-care was essential to call a liver transplant successful [9]. This is possibly due to differences between the two populations, as the previous study had a higher mean age, which is likely to lower their expectations. Moreover, liver transplantation contributes to a significant improvement in the overall health and quality of life of patients as compared with the period before transplantation. It also showed that an increase in the ability to work after liver transplantation was the choice of 87% of patients [12].

Shortage of organs is a significant barrier against organ transplantation and awareness about organ transplantation and donation is an essential factor [15-16]. In our study, results regarding willingness to donate showed that 41% of students would donate, and 18.7% would not. A study from India showed that two-thirds of the medical students said they would donate [17]. And a recent study done in Saudi Arabia found that around 62% of participants favored donating [18]. Awareness level was shown to have a positive effect on the willingness to donate [19-20]. However, knowledge about organ donation and transplantation is not enough to support the decision to donate; motivation plays an important role [21-22].

Regarding organ-donor allocation for liver transplantation, physicians cannot be sure that they have enough organs to save a patient’s life and, thus, two concepts are weighed against each other: the urgency of the case and the prospect of success. Also, this is a challenging procedure from several points of view, medical, ethical, and legal [9]. Multiple studies have shown that active illness negatively impacts post-transplant survival. In contrast with the prospect of success, most patients who are not as ill at the time of transplantation show better post-transplant survival [23-25]. Regarding this issue, we asked the participants if there can be a satisfying balance between the two concepts. Most of the results were close; urgency was chosen more by the preclinical participants (50.7%) while the prospect of success was more dominant in the clinical participants’ answers (66.1%), p = 0.021. This shows that progressing in medical education and gaining more knowledge on prognostic factors affect students’ opinions.

The participants’ opinions on how to handle patients with alcohol-related cirrhosis revealed that 19.4% would reject those patients. This result, which we think is inappropriate, might stem from the perspective that alcoholic individuals are responsible for their illness, as most of them tend to relapse [26-27]. Meanwhile, there are 21% who would always accept those patients on the waiting list; their point of view indicates that all patients should receive treatment equally despite different etiologies [28-29]. In an intermediate point of view, 57.7% said they would accept the patients on the waiting list after having been abstinent for six months, to decrease the possibility of relapse after shorter periods of pretransplant abstinence [30]. In addition, abstinence from alcohol for at least six months is a good gesture showing that the patient is willing to improve his lifestyle in many different aspects.

 Limitations and recommendation

Although this study shows significant findings, it faced several limitations including the limited number of studies in the literature. Also, the survey had no tests of validity and reliability, and it lacked accuracy in measuring the criteria of benefit. Therefore, we recommend future studies to test this questionnaire and validate it. Also, we recommend expanding the medical curriculum to involve more knowledge about the allocation system. 

## Conclusions

This study found that gains in both lifetime and quality of life could be considered as criteria of liver transplant success. Most of the respondents wanted the benefit of liver transplant to be added in the allocation system. However, more studies are needed to define and conceptualize the idea of benefit in liver transplantation.
